# The Immunology of Psoriasis—Current Concepts in Pathogenesis

**DOI:** 10.1007/s12016-024-08991-7

**Published:** 2024-04-20

**Authors:** Izabela Sieminska, Monika Pieniawska, Tomasz M. Grzywa

**Affiliations:** 1 University Centre of Veterinary Medicine, University of Agriculture in Krakow, Krakow, Poland; 2 Institute of Human Genetics, Polish Academy of Sciences, Poznań, Poland; 3 Laboratory of Immunology, Mossakowski Medical Research Institute, Polish Academy of Sciences, Warsaw, Poland; 4 Department of Methodology, Medical University of Warsaw, Warsaw, Poland; 5 The Raymond G. Perelman Center for Cellular and Molecular Therapeutics, Department of Pathology and Laboratory Medicine, Children’s Hospital of Philadelphia, Philadelphia, USA

**Keywords:** Psoriasis, Immune response, Skin inflammation, T_H_17 cells, Keratinocyte

## Abstract

Psoriasis is one of the most common inflammatory skin diseases with a chronic, relapsing-remitting course. The last decades of intense research uncovered a pathological network of interactions between immune cells and other types of cells in the pathogenesis of psoriasis. Emerging evidence indicates that dendritic cells, T_H_17 cells, and keratinocytes constitute a pathogenic triad in psoriasis. Dendritic cells produce TNF-α and IL-23 to promote T cell differentiation toward T_H_17 cells that produce key psoriatic cytokines IL-17, IFN-γ, and IL-22. Their activity results in skin inflammation and activation and hyperproliferation of keratinocytes. In addition, other cells and signaling pathways are implicated in the pathogenesis of psoriasis, including T_H_9 cells, T_H_22 cells, CD8^+^ cytotoxic cells, neutrophils, γδ T cells, and cytokines and chemokines secreted by them. New insights from high-throughput analysis of lesional skin identified novel signaling pathways and cell populations involved in the pathogenesis. These studies not only expanded our knowledge about the mechanisms of immune response and the pathogenesis of psoriasis but also resulted in a revolution in the clinical management of patients with psoriasis. Thus, understanding the mechanisms of immune response in psoriatic inflammation is crucial for further studies, the development of novel therapeutic strategies, and the clinical management of psoriasis patients. The aim of the review was to comprehensively present the dysregulation of immune response in psoriasis with an emphasis on recent findings. Here, we described the role of immune cells, including T cells, B cells, dendritic cells, neutrophils, monocytes, mast cells, and innate lymphoid cells (ILCs), as well as non-immune cells, including keratinocytes, fibroblasts, endothelial cells, and platelets in the initiation, development, and progression of psoriasis.

## Introduction

Psoriasis is a chronic inflammatory skin disease whose prevalence varies from 0.1 to 8% depending on the geographical region and affects more than 125 million people worldwide [1–3]. The clinical features of psoriasis are heterogeneous and include cutaneous as well as systemic manifestations [3]. The most common variant of psoriasis is plaque psoriasis (psoriasis vulgaris) which accounts for more than 80% of cases [1]. Common symptoms include skin itching, burning, and soreness [3]. Moreover, several comorbidities have been associated with psoriasis including psoriatic arthritis, cardiovascular disease, metabolic syndrome, obesity, inflammatory bowel disease, and psychiatric disorders that are associated with systemic inflammation [3, 4].

The etiology of psoriasis is very complex and involves multiple intrinsic and extrinsic factors. Several genetic risk factors, including genes involved in antigen presentation (*HLA-Cw6*), cytokine signaling (*IL12B, IL23R*), interferon signaling, and NF-κB signaling (T*NFAIP3*, *NFKBIA*, *NFKBIZ*, *TNIP1*, and *RELA*) have been identified [3, 5–7]. Psoriasis is characterized by the activation of inflammatory pathways in both the innate and adaptive immune cells leading to the uncontrolled proliferation of keratinocytes (KCs), acanthosis, neovascularization, and potent skin infiltration by immune cells [8]. Over 80% of upregulated genes in psoriatic lesions are associated with activation of KCs and skin infiltration by T cells and macrophages [9]. Moreover, most significantly enriched transcripts are associated with immune response, defense response, and response to wounding [10].

## Immune Response in Psoriasis

Skin is a complex immune organ that protects the organism from infection, enables wound healing, and interacts with the skin microbiome [11–14]. Moreover, it constitutes an important reservoir of immune cells and contains nearly twice the T cells present in the circulation [15]. Thus, disruption of the immune homeostasis may have detrimental effects on the human body and result in a variety of inflammatory diseases.

Psoriasis is characterized by the dysregulation of the cytokine network with multiple self-amplifying feeds accelerating pathogenic circuits. Psoriatic inflammation can be triggered in predisposed individuals by mechanical stress (Koebner phenomenon), air pollutants, sun exposure, drugs, infections, or vaccination [16]. It seems that pattern recognition receptors (PRRs), especially Toll-like receptors (TLRs), are crucial mediators of the response to these triggering factors [17]. Skin injury triggers the release of damage-associated molecular patterns (DAMPs) including dsRNA, ssRNA, and DNA from damaged cells which activate TLRs signaling in different types of cells, including KCs and dendritic cells (DCs) [18, 19]. Activation of TLRs by DAMPs or pathogen-associated molecular patterns (PAMPs) triggers the production of multiple cytokines and initiation of the psoriatic inflammation [19–22].

**Fig. 1:**
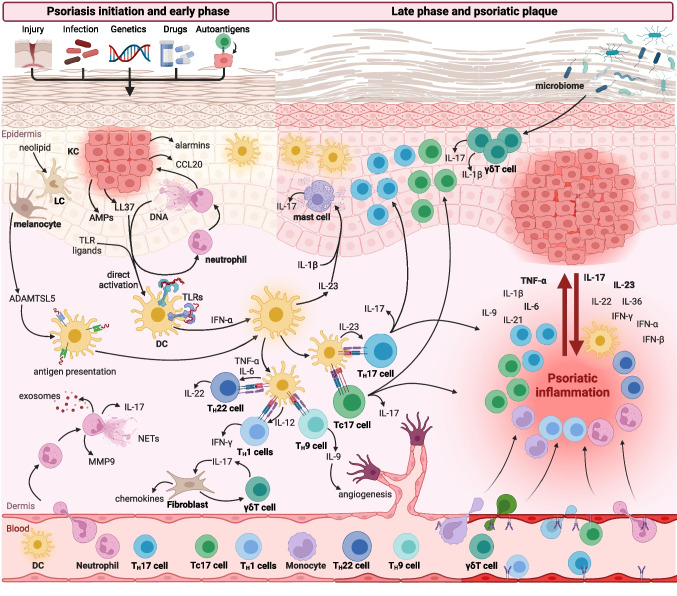
Dysregulation of immune response in psoriasis. Psoriasis may be triggered by different factors, including skin injury (Koebner phenomenon), infection, drugs, and autoantigens in predisposed individuals. In the early phase of psoriasis, neutrophils infiltrate the skin and release neutrophils extracellular traps (NETs), exosomes, metalloproteinase 9 (MMP9), and IL-17. Activated keratinocytes (KCs) have increased proliferative capacity and produce a variety of pro-inflammatory factors, including chemokines, antimicrobial peptides (AMPs), and alarmins. Dendritic cells (DCs) are activated by toll-like receptors (TLRs) ligands and AMPs which initiate T cell immune response. DCs activate a variety of T cells, including IL-17-producing T_H_17 and Tc17 cells, IL-9-producing T_H_9 cells, IFN-γ-producing T_H_1 cells, and IL-22-producing T_H_22 cells. Moreover, DCs and Langerhans cells (LCs) can present autoantigens stimulating autoreactive T cells. The impaired skin microbiome activates γδ T cells that produce IL-17 and IL-1β. In the late phase of inflammation, psoriatic lesions are characterized by the profound infiltration of immune cells, increased concentrations of multiple cytokines and chemokines, and hyperproliferation of KCs. Moreover, increased angiogenesis and endothelial cells activated by psoriatic cytokines facilitate the infiltration of immune cells into inflamed skin. Created with Biorender.com

The first studies suggested that psoriasis is a classical T_H_1 inflammatory disease with IFN-γ as a key mediator of psoriatic inflammation [23]. Further intense studies revealed that it is associated with not only overactivated T_H_1 response but also T_H_17 and T_H_22 responses [24]. In general, proinflammatory cytokines and factors stimulating proliferation in psoriasis are produced mainly by T cells (IL-17, IL-21, IL-22, IFN-γ), DCs (TNF-α, IL-6, IL-20, IL-23, NO), and KCs (antimicrobial peptides (AMPs), IL-20, chemokines). These cells form a key pathogenic loop in psoriasis that involves a triad of IL-23-producing DCs, IL-17-producing T_H_17, and activated KCs (Fig. 1).

### Dysregulation of Cytokine Network Underlies Psoriasis Pathogenesis

Psoriasis has been recognized as a disease of dysregulated cytokine profile for over three decades [26]. Novel methods that enable the assessment of multiple cytokines [27] and transcriptomic genome-wide expression analysis [9, 28] uncovered a profoundly disrupted cytokine expression profile in psoriatic skin as well as a complex network between cells in psoriatic skin (Table 1).

**Table 1:** The role of cytokines and chemokines in psoriasis

**Cytokine**	**Cellular source**	**Level in psoriasis**	**Biological effects in psoriasis**	**Ref.**
IL-1β	Activated macrophages, DCs, KCs, and T cells	↑ lesional skin	- Activates the pro-inflammatory response of KCs	[29–33]
			- Promotes the formation of perivascular DCs clusters	
			- Triggers T_H_17 cells differentiation	
			- Induces proliferation of dermal γδ T cells	
IL-2	DCs, activated T cells, macrophages	↑ lesional skin	- T cells survival factor	[34]
			- Promotes T cells differentiation into effector T cells or memory T cells	
IL-4	Activated T_H_2 cells, basophils, mast cells, ILC2	↓ in psoriatic epidermal T cells	- Promotes T_H_2 immune response	[35–37]
			- Suppresses T_H_1 and T_H_17 responses	
			- Suppresses IL-1β and IL-6 production by KCs	
			- Suppresses IL-23 production by DCs and promotes p35 production	
IL-6	DCs, endothelial cells, KCs, T cells	↑ lesional skin	- Promotes T_H_17 cells differentiation	[38–41]
		↑ serum of psoriasis patients	- Inhibits suppressive functions of Tregs	
			- Induces angiogenesis by upregulating VEGF production	
IL-7	Hair follicle KCs	↑ lesional skin	- Maintains CD4^+^ and CD8^+^ skin-resident memory T cells in the epidermis	[42–44]
		↑ serum of psoriasis patients		
IL-8 (CXCL8)	Neutrophils, KCs	↑ lesional skin	- Stimulates neutrophil infiltration	[41, 45–47]
		↑ serum of psoriasis patients	- Induces KCs hyperproliferation	
			- Stimulates angiogenesis	
IL-9	T_H_9 cells and T_H_22 cells	↑ lesional skin	- Induces IL-17A production by CD4^+^ T cells	[48, 49]
		↑ plasma of psoriasis patients	- Stimulates angiogenesis	
IL-10	T_H_ cells, monocytes, macrophages, and DCs	↓ lesional skin	- Suppresses type 1 inflammation	[50]
		↑ serum of psoriasis patients	- Downregulates IL-8/CXCR2 pathway in epidermal cells	
			- Reduces KCs proliferation	
IL-11	Fibroblasts, epithelial cells	↑ lesional skin	- Reduces T cells infiltration	[51]
			- Polarizes immune response towards type 2 response	
			- Reduces KCs proliferation	
IL-12	Activated DCs, macrophages, monocytes, and B cells	↑ IL-12p40 and IL-12p70 in lesional skin	- Inhibits the invasion of γδT17 cells	[27, 33, 41, 52, 53]
		↑ serum of psoriasis patients	- Induces protective transcriptional program in KCs limiting skin inflammation	
		- No changes or decreased level of IL-12p35	- Induces IFN-γ production by NK cells and T cells	
			- Promotes differentiation of T_H_1 cells	
IL-13	Activated T_H_2 cells, mast cells, and basophils	↑ lesional skin	- Functional role in psoriasis is unclear	[50, 54]
		↑ serum of psoriasis patients		
IL-15	Hair follicle KCs	⟷ serum of psoriasis patients	- Synergizes with IL-23 to induce IL-17F, IL-17A, and IFN-γ production by T cells	[44, 50, 55]
			- Induces T cells infiltration	
			- Stimulates angiogenesis	
IL-16	KCs	↑ serum of psoriasis patients	- Promotes recruitment of CD4^+^ T cells to psoriatic lesions	[56]
IL-17	T_H_17 cells, Tc17 cells, NK cells, γδ T cells, mast cells, neutrophils	↑ IL-17A, IL-17C, and IL-17F in lesional skin	- Increases the level of LL37	[25, 27, 40, 49, 57–61]
			- Promotes secretion of multiple proinflammatory cytokines, including IL-1β, IL-6, GM-CSF, and TNF	
			- Upregulates production of chemokines	
			- Impairs Tregs suppressive functions	
IL-18	KCs	↑ lesional skin	- Promotes infiltration of immune cells in psoriasiform inflammation	[27, 41, 62, 63]
		↑ serum of psoriasis patients	- Upregulates the expression of IL-17 and suppresses the expression of IL-4 in psoriatic skin	
IL-19	KCs	↑ lesional skin	- Upregulates the expression of inflammatory mediators in KCs	[33, 60, 64]
		↑ serum of psoriasis patients	- Synergizes with IL-17A to induce production of β-defensin, IL-23p19, and T_H_17- and neutrophil-attracting chemokines	
IL-20	KCs, leukocytes	↑ lesional skin	- Promotes angiogenesis and chemotaxis	[33, 60]
		↑ serum of psoriasis patients	- Promotes keratinocyte differentiation and activation	
			- Stimulates production of pro-inflammatory cytokines	
IL-21	CD4^+^ T cells, especially T_H_17 cells	↑ lesional skin	- Promotes KCs hyperproliferation	[33, 65–68]
		↑ serum of psoriasis patients	- Promotes and sustains T_H_17 cells differentiation	
			- Inhibits Tregs differentiation	
IL-22	Mast cells, T_H_22 cells, T_H_17 cells, Tc cells, NK-T cells, γδ T cells, DCs, macrophages	↑ lesional skin	- Stimulates KCs hyperproliferation, differentiation, and migration	[33, 58, 60, 69–73]
		↑ serum of psoriasis patients	- Induces the secretion of pro-inflammatory cytokines	
IL-23	Inflammatory monocytes, mature DCs, KCs	↑ lesional skin	- Promotes T_H_17 activation, survival, and pathogenic potential	[53, 74–77]
			- Activates dermal γδ T cells and promotes their expansion	
			- Stimulates antigen presentation by DCs	
			- Triggers IFN-γ production	
			- Promotes immune cells infiltration to the skin	
			- Triggers hyperplasia of KCs	
IL-24	KCs, monocytes	↑ lesional skin	- Induces psoriasis-like inflammation	[60, 78, 79]
IL-25 (IL-17E)	KCs	↑ lesional skin	- Stimulates pro-inflammatory phenotype and proliferation of KCs	[33, 80]
			- Stimulates infiltration of DCs, macrophages, and γδ T cells to the skin	
IL-26	T_H_1 cells, T_H_17 cells, NK cells	↑ lesional skin	- Promotes infiltration of neutrophils and CD4^+^ T cells into the skin	[58, 81, 82]
			- Stimulates angiogenesis	
IL-33	Epithelial cells, KCs, DCs	↑ lesional skin	- Promotes pro-inflammatory phenotype of KCs	[83]
IL-36γ (IL-1FG)	KCs, monocytes, DCs	↑ lesional skin	- Induces the expression of AMPs and matrix metalloproteinases (MMPs) by KCs	[60, 84–86]
			- Regulates the recruitment of inflammatory cells and the expansion IL-17–producing γδ T cells in the skin	
IL-37	Macrophages, effector memory T cells	↓ lesional skin	- Inhibits the production of inflammatory mediators by KCs	[28, 87, 88]
IL-38	KCs	↓ lesional skin	- Suppresses psoriatic inflammation	[89, 90]
		↓ serum of psoriasis patients	- Suppresses pro-inflammatory phenotype of KCs	
			- Suppresses IL-17A production by dermal γδ T cells	
IFN-α	pDCs	↑ lesional skin	- Induces T_H_1 and T_H_17cells and their activation and proliferation	[50, 91–93]
		↑ serum of psoriasis patients	- Activates DCs and KCs	
IFN-β	KCs, pDCs	↑ lesional skin	- Regulates KCs differentiation	[18, 91, 94]
			- Promotes DCs activation and differentiation	
IFN-γ	T cells, NK cells	↑ lesional skin	- Activates KCs	[27, 33, 41, 58, 95]
		↑ serum of psoriasis patients	- Promotes T cells infiltration	
			- Promotes DCs maturation	
TGF-β	Tregs	↑ lesional skin	- Inhibits KCs proliferation	[96–99]
		↑ serum of psoriasis patients	- Promotes T cell infiltration	
TNF-α	Activated T cells, DCs, macrophages, KCs, fibroblasts	↑ lesional skin ↑ serum of psoriasis patients	- Activates T cells, macrophages and DCs	[33, 41, 70, 95, 100–104]
			- Activates KCs triggering hyperproliferation	
			- Synergizes with IL-17A	
			- Promotes secretion of pro-inflammatory cytokines	
			- Induces infiltration of immune cells to the lesional skin	
			- Promotes angiogenesis by inducing VEGF secretion	

**Fig. 2:**
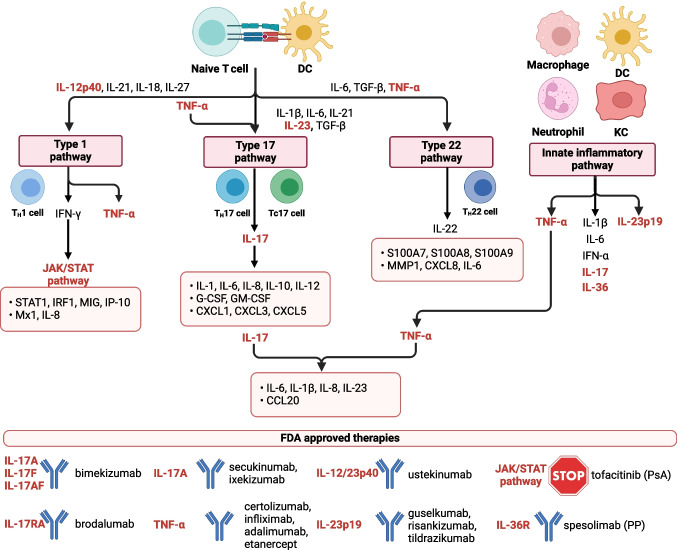
Cytokine network and therapeutic targets in psoriasis. Immune response in psoriasis is associated with the activation of type 1, type 17, and type 22 pathways and innate inflammatory pathways. These signaling pathways induce the expression of different genes involved in the regulation of psoriatic inflammation (orange boxes). Several biological drugs targeting key psoriatic cytokines (indicated in red) or small molecule inhibitors of downstream cytokine signaling were developed and approved by the FDA. PsA, psoriasis arthritis; PP, pustular psoriasis. Created with Biorender.com

Dysregulation of the cytokine profile is associated with overactivation of type 1, type 17, and type 22 pathways as well as innate inflammatory pathways (Fig. 2). TNF-α is the founder cytokine that initiates downstream inflammatory signaling in psoriasis. There are multiple triggers of TNF-α secretion, including skin injury, environmental stimuli, autoantigens, and TLRs agonists [105, 106]. In psoriatic skin, it is produced predominantly by activated T cells and antigen-presenting cells (APCs), including dermal DCs [102–104]. TNF-α synergizes with IFN-γ to induce the expression of chemokines and inflammatory adhesion molecules by endothelial cells which promote the infiltration of immune cells, especially T cells, into the skin [95]. Moreover, TNF-α triggers KCs and DCs to secrete IL-23 [53, 107].

IL-23 is a heterodimer of p19 and p40 subunits. It is a key regulator of the type 17 pathway activating a variety of cells, including T_H_17, Tc17, γδ T cells, and innate lymphoid cells type 3 (ILC3) to produce IL-17 [108]. Moreover, IL-23 is a key cytokine that regulates the survival and pathogenic potential of T_H_17 cells [109–112].

T_H_17 cells orchestrate inflammation via multiple cytokines, especially IL-17. This cytokine regulates immune response to different pathogens and tissue repair processes. However, IL-17 is also implicated in a variety of T_H_17-mediated inflammatory autoimmune diseases [113, 114]. In lesional skin, the level of IL-17 family members, especially IL-17A, IL-17C, and IL-17F, is potently upregulated [25, 40, 80, 115–118]. It is caused by the potent infiltration, activation, and expansion of T_H_17 cells and positive feedback loops amplifying the production of IL-17 [119]. IL-17 exerts a variety of effects in inflamed psoriatic skin, including activation of KCs to produce AMPs, upregulation of ICAM-1 in endothelial cells to promote tissue inflammation and promotion of the infiltration of immune cells. Moreover, IL-17 potentiates inflammation by the induction of multiple pro-inflammatory cytokines and chemokines (Fig. 2) [40, 80, 115–118]. IL-17 synergizes with TNF-α to induce hyperproliferation of KCs, T_H_17-polarized inflammation, and upregulate psoriasis-related genes [61, 118, 120–122]. IL-17 also upregulates the level of psoriasis autoantigens which is potentiated by the vitamin D3 and IL-22 [123].

### Chemokines and Homing Receptors Regulate Immune Cell Trafficking in Psoriasis

Profound infiltration of immune cells into psoriatic skin is caused by, among others, dysregulated chemokine signaling (Table 2). The expression of chemokines and their receptors are regulated by psoriasis-associated cytokines, including IL-17 which upregulates CCL2, CCL7, CCL20, and CXCL1, or IFN-γ which upregulates CXCL9 and CXCL10 [113, 124]. Notably, some of them, including CXCL9 and CXCL10, are upregulated even in non-lesional skin of psoriasis patients which may contribute to the initiation of novel skin lesions [125].

**Table 2:** Chemokine and their receptors in psoriasis

**Chemokine**	**Receptor**	**Biological effects in psoriasis**	**Level in psoriasis**	**Ref.**
CCL2/MCP-1	CCR2, CCR4	- DCs and Langerhans cells chemotaxis to the skin	↑ CCR2 in lesional skin	[27, 126, 127]
		- CCR4 is a skin-homing receptor	↑ CCR4 in T cells in peripheral blood and psoriatic skin lesions	
			↑ CCL2 in lesional skin and serum	
			↑ CCL2 in KCs by TNF-α and IFN-γ	
CCL3/MIP-1α	CCR1, CCR5	- T_H_1 cells, DCs, and monocytes chemotaxis to the skin	↑ CCL3 in lesional skin	[27, 128]
		- CCR5 regulates lymphocyte detention in the dermis	↑ CCR5 in lesional skin	
			↑ CCR5^+^ T cells and macrophages in the dermis of lesional skin	
CCL4/MIP-1β	CCR1, CCR5	- T_H_1 cells, DCs, and monocytes chemotaxis to the skin	↑ CCL4 in lesional skin	[27, 128, 129]
CCL5/RANTES	CCR1, CCR3, CCR5	- T_H_1 cells and monocytes chemotaxis to the skin	↑ CCL5 in lesional skin	[27, 47, 128, 130]
			↑ CCL5 in the KCs in lesional skin	
			↑CCL5 by IFN-α	
CCL19/MIP-3 β	CCR7	- T cells and DCs chemotaxis to the lymph nodes, regulation of dermal lymphoid-like tissue	↑ CCL19 in lesional skin	[131]
			↑ CCR7 in lesional skin	
CCL20/MIP-3α	CCR6	- T_H_17 cells, γδ T cells, DCs, and LCs chemotaxis to the skin	↑ CCL20 in lesional skin	[127, 132–134]
			↑ CCL20 in KCs by IFN-γ, IL-17A, and IL-22	
			↑ CCL20 in KCs, melanocytes, and dermal endothelial cells by TNF-α and IL-1β	
			↑ CCR6^+^ T cells in lesional skin	
			↑ CCR6 by T_H_17 cytokines	
CCL21/SLC	CCR7	- T cells and DCs chemotaxis to the lymph nodes	↓ CCL21^+^ vessels in psoriatic skin	[135]
CCL18/PARC	CCR8	- CD4^+^ and CD8^+^ T cells chemotaxis	↑ CCL18 in lesional skin	[136]
CCL27/CTACK	CCR10	- CLA^+^ T cells chemotaxis	↓ CCL27 in lesional skin	[129, 137, 138]
			↑ CCL27 in serum of psoriasis patients	
			↑ CCR10 in T cells in psoriatic lesions	
			↑ CCL27 in KCs by TNF-α and IL-1β	
CXCL1/GRO-α	CXCR2	- Neutrophils chemotaxis to the skin	↑ CXCR2 in psoriatic KCs	[139]
CXCL5/ENA78	CXCR2	- Neutrophils chemotaxis to the skin	↑CXCL5 in the serum of psoriasis patients	[139, 140]
			↑ CXCR2 in psoriatic KCs	
CXCL9/MIG	CXCR3	- T cells chemotaxis	↑ CXCL9 in lesional skin	[95, 125, 141]
			↑ CXCR3 in lesional skin	
			↑ CXCR3 in epidermal T cells, macrophages, and pDCs in lesional skin	
CXCL10/IP-10	CXCR3	- T cells chemotaxis	↑ CXCL10 in lesional skin, especially in KCs	[27, 47, 95, 125]
CXCL11/I-TAC	CXCR3	- T cells chemotaxis	↑ CXCL11 in lesional skin	[125, 130]
			↑ CXCL11 by IFN-α	
CXCL12/SDF-1	CXCR4, CXCR7	- Neutrophils, monocytes, T cells, and DCs chemotaxis	↑ SDF-1 in lesional skin	[142]
			↑ CXCR4 in lesional skin	
CX3CL1	CX3CR1	- Migration and adhesion of leukocytes	↑ CX3CL1 in endothelial cells and KCs in lesional skin	[143]
			↑ CX3CL1 by TNF-α and IFN-γ	
			↑ CX3CR1 in T cells in lesional skin	

In psoriasis, T cells infiltrating lesional skin have substantially upregulated expression of chemokine receptors, including a skin-homing receptor, CCR4, and CCR6 [127]. Moreover, there is an increased fraction of CCR4^+^ and CCR6^+^ T cells in peripheral blood [70]. Due to increased expression of chemokine receptors, skin-homing T cells of psoriasis patients respond to lower concentrations of chemokines, including CCL20, and exhibit stronger chemotactic responses compared to T cells of healthy individuals [132].

## Immune Cells in Psoriasis

**Fig. 3:**
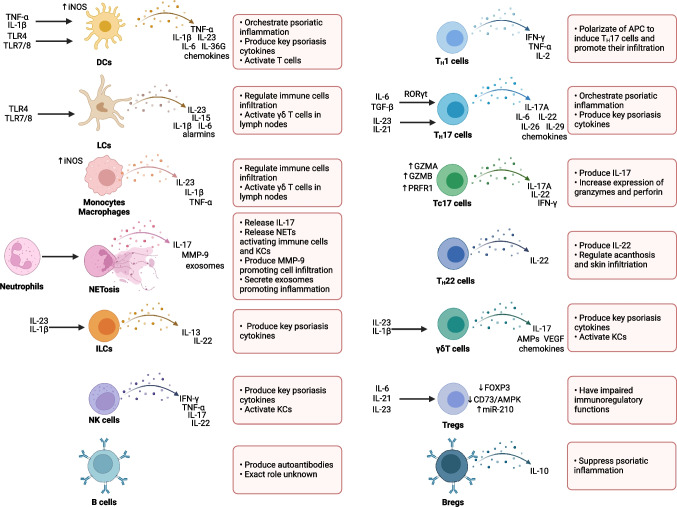
The role of immune cells in psoriasis. The key populations of immune cells and their functions in the regulation of psoriatic inflammation. Created with Biorender.com

Multiple types of immune cells regulate the initiation, maintenance, and progression of psoriatic inflammation. T cells, especially T_H_17 cells, together with DCs are the main players in the pathogenesis of psoriasis. Nonetheless, a variety of other types of immune cells, including neutrophils, monocytes, macrophages, mast cells, and ILCs participate in the pathogenesis of psoriasis (Fig. 3). In general, innate immune cells, especially neutrophils, are key cells in the early phases of psoriasis development while T cell-dominated adaptive inflammation is a feature of stable plaques in the later phases [144].

### The Role of Neutrophils

Neutrophils are a heterogeneous innate immune cell population that can either suppress or enhance immune response [145]. In psoriasis, neutrophils participate in the initiation of the disease and early phases of progression of psoriatic inflammation [146, 147]. Early lesions and prepsoriatic skin adjacent to active lesions are characterized by the potent infiltration of CD15^+^ neutrophils [148]. Chronic lesions are also infiltrated by neutrophils, especially CD15^pos^CD10^pos^ and CD15^pos^CD10^neg^ neutrophils [146] Neutrophils are also enriched in the peripheral blood of psoriatic patients and are in a pre-activated state [149]. They are chemoattracted to the inflamed psoriatic skin mainly by IL-17E and CXCL8 [149]. Additionally, neutrophils release IL-17A and extracellular vesicles that induce the expression of proinflammatory cytokines and chemokines in KCs which enhance their migratory phenotype and promote infiltration into the skin [150, 151]. Notably, infiltrating neutrophils overexpress matrix metallopeptidase (MMP)-9 that activates vascular endothelial cells, enhances vascular permeability, and promotes CD4^+^ T cells infiltration [152].

In psoriatic lesions, IL-17^+^ neutrophils together with IL-17^+^ mast cells (MCs) are found in higher densities than IL-17^+^ T cells [152]. It seems that neutrophils do not express IL-17A, but can accumulate it [61] and release it during the formation of neutrophil extracellular traps (NETs) [152]. Indeed, neutrophils in psoriasis frequently undergo NETosis, even without any stimulation [152]. Moreover, they can be triggered and activated in psoriatic skin by different types of cells, especially by KCs [151, 153]. DNA- and RNA-containing NETs are highly abundant in the psoriatic skin as well as peripheral blood of psoriasis patients [152, 154]. Notably, NETosis in peripheral blood correlates with the clinical severity of psoriasis patients [154]. Moreover, low density granulocytes circulating in psoriasis patients blood seems to be more sensitive to cutaneus stimuli cousing their release of NETs then conventional neutrophils, leading to skin pathology [155]. Released nucleic acids form complexes with LL37 which triggers NETs and cytokine release by neutrophils in TLR8/TLR13- or TLR4/IL-36R-mediated mechanisms [156, 157]. Moreover, the release of NETs and complexes of LL37 with DNA/RNA activates other types of immune cells, promotes an inflammatory response in KCs, and induces IL-17 secretion by T cells [146, 154, 157].

### The Role of Monocytes and Macrophages

In murine models, psoriatic inflammation is associated with the expansion of activated monocytes and macrophages in lesional skin and draining lymph nodes [158, 159]. These activated macrophages are a key source of TNF-α [76, 158]. Likewise, the lesional skin of psoriasis patients is characterized by the accumulation of activated macrophages in both the epidermis and dermis, especially in the late phase of psoriatic inflammation [76, 160].

Macrophages in psoriasis are “classically activated” by IFN-γ and produce IL-23p19, IL-12/23p40, iNOS, and TNF-α [161]. Moreover, IL-17 activates the pro-inflammatory phenotype of monocytes/macrophages and sensitizes them toward pathogen-derived TLR4 ligands [162]. Monocytes are attracted to inflamed psoriatic skin by different chemokines and cytokines [76]. Moreover, there is an expansion of immunomodulatory immature myeloid cells called myeloid-derived suppressor cells (MDSCs), especially monocytic MDSCs (M-MDSCs), in the peripheral blood and skin lesions of psoriatic patients that correlate with the clinical severity of psoriasis [165]. However, the role of MDSCs in the pathogenesis of psoriasis is unknown.

### The Role of Mast Cells

Mast cells are key effector cells in acute allergic reactions and inflammatory diseases [166, 167]. Moreover, their expansion and activation contribute to psoriatic inflammation [168]. In psoriasis, mast cells express and release IL-17 and IL-22 [152]. Notably, analysis of psoriatic skin biopsies revealed that mast cells constitute most of the IL-17-containing and IL-22-containing cells in psoriatic lesions [152]. Psoriatic mast cells degranulate and form mast cell-extracellular traps (MCETosis) after stimulation with IL-1β and IL-23 which trigger the release of IL-17 [72, 152]. Notably, mechanical injury induces a pro-inflammatory response of mast cells which secrete tryptase, IL-6, IL-17, IL-18, and IL-36γ and activate KCs initiating psoriatic inflammation in the Koebner phenomenon [169].

### The Role of Innate Lymphoid Cells

NK cells and NK-T cells are rare in lesional psoriasis skin [170]. However, their numbers significantly increase in the late-phase lesions [171]. In peripheral blood of psoriasis patients, the frequency of NK cells and NK-T cells is decreased, however, with a relative increase in NK-T17 cells [173, 174]. Despite their low numbers in psoriatic skin, NK cells produce high amounts of IFN-γ, TNF-α, IL-17A, and IL-22 [170, 172, 175]. Moreover, NK cells upregulate the expression of MHC class II proteins, ICAM-1, CXCL10, and CCL5 in psoriatic KCs [172]. It was suggested that also other innate lymphoid cells (ILC), including ILC2 and ILC3 participate in the pathogenesis of psoriasis [164, 176, 177]. However, the exact role of ILC in psoriasis patients remains to be determined.

### The Role of Dendritic Cells

DCs are a major type of leukocytes in psoriatic lesions with numbers exceeding the number of T cells [104]. The main role of DCs in psoriasis is to orchestrate the immune response of T cells. Psoriatic DCs strongly induce both T_H_17 and T_H_1 T cells [178]. Notably, some of the T cells activated by psoriatic DCs simultaneously produce both IFN-γ and IL-17, which is not the case for healthy skin dermal DCs [178].

DCs in psoriatic lesions are divided into two main groups—CD11c^+^ BDCA-1^+^ resident DCs that are found also in healthy skin, and CD11c^+^ BDCA-1^−^ inflammatory DCs that are more common in psoriasis lesional skin and are immature DCs that produce inflammatory cytokines [178]. These inflammatory DCs also include TNF-inducible NO synthase (iNOS)-producing DCs (TipDCs) [53, 179]. TipDCs are greatly increased in the lesional psoriatic skin [104, 180]. A similar population of DCs, that express IL-23p19, TNF-a, and iNOS, is called slan (6-sulfo LacNAc) DCs (slanDCs) and contributes to psoriasis by producing IL-17 and IL-22 [180, 181]. SlanDCs are enriched in lesional skin and are highly responsive to the TLR7 and TLR8 agonists as well as to complexes of self-RNA and LL37 [180, 181].

While in healthy skin DCs are restricted to the dermis, nearly half of DCs in psoriatic skin are located in the epidermis [104, 160, 182]. The population of epidermal DCs (eDCs) differs from a dermal counterpart and includes previously defined TipDCs, slan-DCs, and other types of DCs [180]. In general, eDCs express multiple genes regulating the activation of KCs and T cells as well as the recruitment of neutrophils, including IL-1β, IL-6, IL-8, CXCL1, and CCL17 [180].

Plasmacytoid dendritic cells (pDCs) are a rare population of DCs responsible for innate antiviral immunity [183]. These DCs are absent in healthy skin but accumulate in the skin of psoriasis patients [91]. Notably, they were identified to initiate psoriatic inflammation [91]. pDCs are activated by self-RNA-LL37 and self-DNA-LL37 complexes as well as other ligands via TLR signaling, especially TLR9 and TLR7. Complexes consisting of DNA or RNA and LL37 are abundant in lesional skin [19, 106]. In response to stimulation through TLR7 and TLR9, pDCs produce IFN-α that activates and induces expansion of T cells [91]. In the murine xenograft model of human psoriasis, inhibition of IFN-α completely prevented the development of T cell-dependent psoriasis [91].

pDCs are attracted to the early psoriatic lesions by chemerin, a chemotactic factor secreted by fibroblasts, mast cells, and endothelial cells [148]. Moreover, pDCs chemotaxis is promoted by VEGF-A-producing dermal IL-17A^+^ γδ T cells from the psoriatic skin [184]. Besides pDCs, conventional DCs (cDCs) participate in the induction and maintenance of psoriatic inflammation by the production of IL-23 [185, 186].

Another subset of DCs, Langerhans cells (LCs), which is a skin-resident population, contributes to the initial phase of psoriasis [186]. Activated psoriatic KCs produce bone morphogenetic protein 7 (BMP7) that promotes the differentiation of progenitor cells into inflammatory LCs [187]. LCs produce IL-6 and IL-23 leading to the infiltration of γδ T cells and CD4^+^ T cells into the skin [188, 189]. Notably, LCs migrate to the draining lymph nodes to induce expansion of IL-17A-producing γδ T cells [188]. In psoriasis, LCs have upregulated expression of S100A8 and S100A9 alarmins, increased production of IL-15, and produce IL-23 and IL-1β in response to TLR4 and TLR7/8 stimulation [180].

### The Role of T cells

T cells are a crucial element of skin immunity [12]. There are approximately 20 billion antigen-experienced memory T cells in the skin of healthy adults [190]. Notably, the number of T cells is even up to 100 times higher in active psoriatic lesions than in healthy skin [191]. In psoriasis, activated T cells preferentially accumulate and expand in the epidermis [191, 193]. Accordingly, the substantial upregulation of IL-17A, IL-22, and IFN-γ is observed in epidermal T cells and to a lesser extent in their dermal counterparts [191]. Notably, while in healthy skin IL-17A is produced mainly by CD4^+^ T cells and IFN-γ is produced predominantly by CD8^+^ T cells, in psoriasis, both CD4^+^ T cells and CD8^+^ T cells co-produce both IL-17A and IFN-γ [164, 194].

#### T_H_ Cells

CD4^+^ T helper (T_H_) cells orchestrate skin inflammation in psoriasis [195]. Both skin-infiltrating and circulating T_H_ cells in psoriasis are predominantly T_H_1, T_H_9, T_H_17, and T_H_22 cells [70, 197].

#### T_H_1 Cells

Psoriasis was at first considered a typical T_H_1 inflammatory disease [23]. It was supported by the fact that epidermal T cells in psoriasis strongly produce type 1 cytokines, including IFN-γ, IL-2, and TNF-α [36]. T_H_1 cells and their main cytokine IFN-γ are increased in psoriatic lesions and peripheral blood of psoriasis patients [70]. Moreover, circulating CD4^+^ T cells in psoriasis are enriched in TNF-α^+^ cells compared to healthy controls [70].

#### T_H_17 Cells

The main factors regulating differentiation from naïve T cells toward T_H_17 lineage are IL-6 and TGF-β which activate the orphan nuclear receptor RORγt inducing the T_H_17 differentiation program [198, 199]. Both cytokines are upregulated in psoriasis (Table 1). Indeed, T_H_17 cells are substantially enriched in the circulation of psoriasis patients [70, 200]. Moreover, T_H_17 cells infiltrate and accumulate in psoriasis skin lesions and are predominantly localized in the dermis [201, 202]. It was found that 3–15% of T cells infiltrating psoriatic skin lesions produce IL-17A [25]. Notably, infiltrating T_H_17 cells accelerate further T_H_17 cells infiltration and expansion via the production of IL-6 in the dermis of psoriatic skin [39] as well as induction of CCL20 production by KCs [134]. Secreted IL-6 induces RORc, IL-17, and IL-23R expression supporting T_H_17 differentiation [68, 74]. The polarization of psoriatic inflammation toward the T_H_17 response is potentiated by IFN-γ produced by T_H_1 which reprograms APCs to produce IL-1, IL-23, and CCL20 [201, 203]. IL-23 produced by activated DCs supports T cell differentiation to T_H_17 cells and their expansion and survival [68, 74]. Thus, T_H_1 and T_H_17 colocalize in the pathological inflammatory environment of psoriasis and create a self-amplifying pathogenic loop. Moreover, cutaneous *Staphylococcus aureus* which is enriched in psoriatic and *Streptococcal pyogenes* that is associated with the onset of some types of psoriasis was identified to trigger a T_H_17 polarization of CD4^+^ T cells [204, 205].

T_H_17 cells are activated by factors that are upregulated in the psoriatic milieu, including IL-23, as well as IL-21 in an autocrine manner [68, 74]. T_H_17 cells express and secrete a variety of pro-inflammatory cytokines and chemokines that support psoriatic inflammation (Fig. 3) [198]. In lesional skin, IL-17F^+^ IFN-γ^+^ cells are the largest subset of T17 T cells and have a high expression of IL-1β, CSF-2, LTA, IL-24, and IL-34 [206].

#### T_H_22 Cells

It was demonstrated that IL-17 and IL-22 can be co-expressed by T_H_17 cells and synergize in the induction of the expression of AMPs by KCs in vitro [208]. However, a meta-analysis of healthy and psoriatic lesional skin transcriptome did not support the existence of dual-secreting IL-17A/IL-22 T_H_17 cells [209]. Furthermore, analysis of circulating immune cells in psoriasis confirmed that most of the T_H_17 cells do not co-express IL-17 and IL-22 [70]. Indeed, further studies revealed that a subpopulation of T cells producing IL-22 is distinct from T_H_17 cells and was named T_H_22 [71, 210]. These cells are crucial in the maintenance of skin homeostasis and contribute to different pathologies, including psoriasis [71, 196, 211]. The frequency of circulating T_H_22 cells is increased in psoriasis and correlates with the clinical severity [70, 73]. Moreover, T_H_22 cells infiltrate and accumulate in lesional psoriatic skin [202]. IL-22 produced by T_H_22 cells mediates acanthosis and immune cell infiltration induced by IL-23 [69]. Notably, T_H_22 cells are detected in the epidermis of resolved lesions after several years of treatment and form a disease memory in clinically healed psoriasis [191]. Thus, they are a promising therapeutic target.

#### T_H_9 Cells

IL-9-secreting T_H_9 cells are skin-tropic or skin-resident and produce TNF-α and granzyme B [212]. Moreover, T_H_9 cells induce the expression of multiple pro-inflammatory cytokines, including IFN-γ, IL-9, IL-13, and IL-17 [212]. Notably, T_H_9 cells are enriched in psoriatic skin lesions over three times compared to healthy skin [212]. Their main role in psoriasis is associated with the stimulation of angiogenesis via IL-9 secretion and potentiation of T_H_17 inflammation [48].

#### T_H_2 Cells

Unlike other populations of T_H_ cells, the frequency of T_H_2 cells and levels of type 2 cytokines (IL-4 and IL-10) are decreased in psoriasis [36]. IL-4 suppresses the production of IL-23 by DCs while promoting the expression of IL-12p70 [213]. Thus, it impairs the induction and maintenance of pathogenic T_H_17 cell-mediated inflammation. Moreover, it suppresses the secretion of IL-1β and IL-6 and the expression of β-defensin 2 by psoriatic epidermal cells [214]. Due to its protective role in psoriatic inflammation, IL-4 therapy was proposed as a promising therapeutic strategy [35, 213].

#### T_FH_ Cells

T follicular help (T_FH_) cells regulate humoral response by enabling the formation of the germinal center with B cells and regulating their maturation [215]. Despite psoriasis is not directly associated with humoral autoimmunity, the frequency of circulating T_FH_ cells is increased in psoriatic patients [216]. Moreover, T_FH_ cells infiltrate and accumulate in psoriasis lesions [216]. Notably, T_FH_ cells in psoriasis are activated and have upregulated IL-21, IL-17, and IFN-γ production [216, 217]. Nonetheless, the mechanistic role of T_FH_ cells in the pathogenesis of psoriasis is unclear.

#### CD8^+^Tc Cells

CD8^+^ cytotoxic T (Tc) cells are key effectors of the immune response against infection or cancer. Moreover, they are implicated in autoimmunity [219]. Tc cells, including Tc17 cells, are enriched in the circulation of psoriasis patients and potently infiltrate psoriatic lesions, especially the epidermis of lesional skin [194, 220]. CD8^+^ Tc cells are also a potent source of pro-inflammatory cytokines, including IL-17, IL-22, and IFN-γ in psoriatic lesions [194, 220]. Moreover, epidermal psoriatic CD8^+^ Tc cells have increased expression of granzyme A, granzyme B, and perforin 1, key proteins responsible for the cytotoxic activity of CD8^+^ T cells [194, 220]. The IL-17A-producing population of CD8^+^ T cells contains not only conventional T cells but also innate CD8^+^ mucosa-associated invariant T cell (MAIT cell) that contributes to psoriatic inflammation [221]. In mice, depletion of CD8^+^ Tc cells completely prevented the development of psoriasis [222]. Therefore, Tc cells together with T_H_ cells are critically involved in the induction and execution of psoriatic inflammation.

#### γδ T Cells

Γδ T cells are selectively enriched in peripheral tissues, including skin, and can produce large amounts of cytokines in a short time [223]. Dermal γδ T cells express IL-23R, IL-17R, RORγt, and a variety of chemokine receptors, including CCR1, CCR2, CCR4, CCR5, CCR6, CXCR3, and CXCR4 [75, 224].

Emerging evidence suggests the pathogenic role of γδ T cells in psoriasis [225]. The frequency of γδ T cells in the dermis is substantially increased in patients with psoriasis [75]. Expansion and activation of γδ T cells in psoriatic lesions are stimulated by IL-1β via IL-1R [31].

In psoriatic skin, γδ T cells robustly produce proinflammatory cytokines, including IL-17, as well as proinflammatory chemokines CCL3, CCL4, CCL5, and CXCL8. Albeit, γδ T cells constitute about 1% of T cells in active psoriatic skin and are not observed in all resolved psoriatic lesions [226], their production of IL-17 is essential for psoriasis inflammation [31, 75, 119, 224]. Production of IL-17 by γδ T cells is induced by IL-1β and IL-23 and by IL-17-activated dermal fibroblast [31, 119]. Moreover, mediators secreted by γδ T cells activate KCs to produce multiple psoriasis-associated mediators, including NF-α, IL-6, CXCL9, CXCL10, and AMPs [227].

#### T Regulatory Cells (Tregs)

The frequency of Tregs in peripheral blood or lesional psoriatic skin compared to healthy individuals varies depending on the study [228]. Nonetheless, it is well-established that the immunoregulatory functions of Tregs are severely impaired in psoriasis [229]. Tregs isolated from lesional skin or peripheral blood of psoriasis patients fail to suppress effector T cell proliferation, in contrast to Tregs isolated from healthy individuals [229, 230]. Notably, in psoriasis Tregs not only fail to suppress the immune response in psoriasis but also actively contribute to psoriatic inflammation by the production of IL-17 [200].

Interestingly, obesity, especially long-chain FFAs, causes Treg loss in the skin and simultaneously increases IL-17A+ γδ T cells by reducing PPARγ+ skin Treg cells and a corresponding loss of control over IL-17A+ γδ T cell-mediated inflammation. Therefore, obesity plays a key role in the development of psoriasis [231].

Several factors impair the immunoregulatory functions of Tregs, including IL-6, IL-21, and IL-23 which are potently enriched in the psoriatic milieu [39, 59, 230]. Inhibition of STAT3, a downstream protein of these cytokines, partially restores suppressive properties of Tregs resulting in the inhibited production of pro-inflammatory cytokines, including IFN-γ, TNF-α, and IL-17, by psoriatic T cells [230]. The mechanisms of impaired immunoregulatory Tregs functions in psoriasis are associated with downregulated CD73/AMPK signaling pathway [232] and an increased level of miR-210 that downregulates FOXP3, a key regulator of the Tregs transcriptional program [233]. However, this phenomenon is most probably much more complex and requires further studies to identify pathways that may be modulated to restore Tregs functions in psoriasis.

#### Memory T Cells

Human skin contains four main populations of memory T cells, CD69^+^CD103^−^ and CD69^+^CD103^+^ non-recirculating resident memory T cells (T_RM_), CCR7^+^/L-selectin^+^ central memory T cells (T_CM_), and CCR7^+^/L-selectin^−^ migratory memory T cells (T_MM_) [234]. T_RM_ cells have increased expression of adhesion molecules and produce a variety of pro-inflammatory and regulatory cytokines [235]. Notably, skin T_RM_ cells are long-lived and, in mice, persist for over a year even in the absence of local antigen presentation [236].

In psoriasis, approximately half of epidermal CD8^+^ Tc cells in the psoriatic skin co-express T_RM_ cell markers CD103 and CD49a and are profoundly enriched compared to healthy skin [191, 237, 238]. The density of infiltrating CD8^+^ T_RM_ cells in the dermis correlates with the thickness of the psoriatic epidermis [239]. CD8^+^ T_RM_ cells in psoriatic skin are an important source of IFN-γ, IL-17A, and IL-22 [191, 237, 238]. CD8^+^ T_RM_ cells can be divided based on the expression of PD-1 into two groups. In psoriasis, PD-1^+^ CD8^+^ T_RM_ cells preferentially expressed IL-23R and produce IL-17A, while PD-1^−^ CD8^+^ T_RM_ cells are a source of IFN-γ [239]. Unlike CD8^+^ Tc cells, only a small fraction of CD4^+^ T_H_ cells express CD103, a marker of T_RM_ cells [237].

T_RM_ cells are a crucial population of immune cells from the clinical point of view since they are key cells that evoke the recurrence of psoriasis after therapy. Psoriatic lesions are often recurrent in the same sites [240], which is caused by the “lesional memory” phenomenon in which T_RM_ cells play a pivotal role [241]. Both IL-17A-producing CD8^+^ T cells and IL-22-producing CD4^+^ T cells form a psoriasis disease–localized memory in the skin even 6 years after the beginning of the successful anti-TNF-α therapy [191, 226]. The latest clinical trial with antibodies against IL-17A and IL-17F (bimekizumab) showed cessation of symptoms in patients with psoriatic arthritis and an inadequate response or intolerance to TNF-α inhibitors [242].

Similarly, a small number of T cells, including T_RM_ cells is also detected after 24 weeks of treatment with anti-IL-17A therapeutics [243]. Further analysis revealed that the IL-17A-producing CD8^+^ T_RM_ cells are associated with early relapse of psoriasis after therapy [239]. In addition to memory αβ T cells, studies in mice identified a subset of IL-17-producing γδ T cells that are long-lived, persist in the skin after psoriasis resolution, and are capable of establishing memory population in the psoriatic skin [244]. Therefore, there is a great clinical interest in the development of therapies targeting T cells in psoriasis skin that constitute immunological memory to prevent disease relapse.

#### T Cells Autoantigens

Besides the initial activation of innate immunity, psoriasis may be also triggered by the initiation of the adaptive immune response. ADAMTS-like protein 5 (ADAMTSL5), a melanocyte protein, was identified as an autoantigen in psoriasis presented by HLA-C*06:02, the main psoriasis risk gene [105, 245]. Activation of autoreactive T cells by ADAMTSL5 triggers a T_H_17 phenotype with strong IL-17A and IFN-γ production [105]. These autoreactive T cells are present in the peripheral blood of two-thirds of psoriasis patients [105].

The antimicrobial peptide cathelicidin (LL37) has been recognized as another autoantigen by T cells in peripheral blood in about half of psoriasis patients [246]. Circulating T cells in psoriasis patients triggered by LL37 produce IFN-γ, IL-17, IL-22, and CXCL8 as well as upregulate perforins and granzyme B [246]. These autoreactive T cells express skin-homing receptors and are detected in lesional psoriatic skin [246]. Notably, LL37 also regulates the fate of non-autoreactive T cells. It suppresses T_H_1 differentiation and promotes T_H_17 differentiation and survival of non-LL37-specific T cells [247]. To exert its effects, LL37 needs to be present during antigen presentation and the T cell activation process, which is indeed a case in vivo since LL37 can be released in the lymph nodes by neutrophils [247]. Both ADAMTSL5 and LL37 are enriched in psoriatic lesional skin, and their levels are upregulated by IL-17 and TNF-α [246, 248].

Another identified autoantigen in psoriasis is the neolipid antigen produced by phospholipase A_2_ (PLA_2_) [249]. PLA2G4D, a cytosolic PLA_2_ group IVD, is highly expressed in psoriatic lesions, especially by the mast cells in the dermis and KCs, while it is not detected in healthy skin [249]. PLA2G4D generates ligands for CD1a from phospholipids in plasma membranes [249, 250]. Notably, mast cells not only produce CD1a ligands but also secrete them in the extracellular vesicles delivering them to the APCs [249]. Several types of cells can present lipid antigens with CD1a; however, Langerhans cells in the skin are crucial in this process [251]. A subset of T cells recognizes lipid antigens presented by CD1a [252]. These CD1a autoreactive T cells are enriched in the peripheral blood and accumulate in the lesional skin of psoriasis patients [249]. In response to CD1a ligands, they produce high amounts of IFN-γ, IL-17A, and IL-22, contributing to psoriatic inflammation [249].

Additionally, T cell receptor (TCR) sequencing in psoriasis revealed that T cell clones that are expanded in psoriasis are detectable also in the non-skin-homing (CLA^−^) population [253]. Thus, they may recognize autoantigens that are not exclusively expressed or located in the skin [253]. However, sequencing of TCR revealed that T cell response in the psoriatic skin is highly polyclonal [254], which excludes one potent autoantigen in psoriasis and suggests a more systemic character of inflammation.

### The Role of B Cells

Despite the recent progress in the studies on the role of skin-associated B cells in the maintenance of skin homeostasis and regulation of inflammatory response [255], the role of B cells in psoriasis has been neglected so far. Nonetheless, psoriasis patients have an increased fraction of B cells, including activated B cells in peripheral blood which correlates with the clinical severity of psoriasis [217, 256]. Moreover, the frequency of B cells is also increased in psoriatic lesional skin [257]. Besides autoreactive T cells, several autoantigens for antibodies were proposed, including heterogeneous nuclear ribonucleoprotein A1 (hnRNP-A1), keratin 13, and Rab coupling protein isoform 3 (FLJ00294) (RAB11FIP1) [258, 259]. Moreover, a group of psoriasis patients has IgG autoantibodies against LL-37 and ADAMTS-L5 [260]. However, the mechanistic role of B cells and autoantibodies in the regulation of psoriatic inflammation remains unknown.

Some evidence of the role of B cells in psoriasis was derived from murine models of psoriasis. Regulatory B cells (Bregs) that suppress the immune response by secretion of IL-10 were found to potently suppress psoriatic inflammation by promoting Tregs expansion while inhibiting T_H_17 cells differentiation [261]. In mice, splenic Bregs enter circulation and migrate to lymph nodes suppressing the production of IFN-γ and IL-17 by lymphocytes in draining lymph nodes and inflamed skin [262]. Nonetheless, studies in psoriasis patients are required to determine the role of Bregs in the pathogenesis of psoriasis.

## Non-Immune Cells Regulating Immunity in Psoriasis

Activation of immune cells in the psoriatic milieu affects all cells present in the skin. Importantly, these cells are not only victims but also actively participate in the amplification of psoriatic inflammation. Here, we described the role of KCs, key non-immune cells, as well as fibroblast, endothelial cells, and platelets in psoriasis.

### The Role of Keratinocytes

KCs constitute about 90% of epidermal cells and are crucial non-immune cells that orchestrate psoriasis initiation and progression [263–265]. Activated KCs contribute to the pathogenesis of psoriasis via two main mechanisms. Firstly, psoriatic KCs have increased proliferative activity which results in epidermal hyperplasia, which is a hallmark of psoriatic plaques. Psoriatic skin is characterized by the dysregulated differentiation of KCs and enrichment of differentiated KCs [164]. Secondly, KCs are a crucial source of multiple immune mediators, including AMPs, chemokines, cytokines, and S100 alarmins (S100A7, S100A8, S100A9) that potentiate inflammation [164, 182, 206]. One of the crucial chemokines produced in a large amount by psoriatic KCs is CCL20 which chemoattracts IL-17-producing CCR6^+^ T_H_17 cells and γδ T cells [132, 266–269].

KCs are crucial responders to psoriatic cytokines, including IL-17, IL-22, IL-36, and IFN-γ [28, 265, 270–272]. In response to activation with these cytokines, KCs upregulate the expression of alarmins and AMPs [208]. Additionally, IL-17A induces the expression and release of IL-25 from KCs [80]. IL-25 in an autocrine manner induces a pro-inflammatory phenotype and hyperproliferation of KCs which is mediated by the activation of STAT3 [80]. Moreover, IFN-γ produced by activated immune cells primes KCs by rapid and long-lasting suppression of miRNA-149, a suppressor of the inflammatory response [273, 274]. In addition to cytokines, KCs are activated in psoriasis by DAMPs [18, 275]. dsRNA-LL37 complexes trigger IFN-β production in KCs which promotes activation and maturation of DCs that in turn drive T cell response [18]. Moreover, enhanced production of highly charged polyamines by psoriatic KCs promotes self-RNA sensing by DCs via TLR signaling [276].

Psoriatic KCs have impaired several self-regulatory pathways. For instance, cholecystokinin (CCK), a peptide hormone that suppresses KC inflammatory response, is potently decreased in lesional psoriatic skin [277]. Moreover, KCs in psoriasis exhibit increased reactivity to various cytokines. KCs from the psoriatic lesions more robustly respond to the TNF-α and IL-17 by the IL-23 production that KCs from healthy patients [120]. Moreover, some cytokines in the psoriatic milieu, including IL-25 and IL-29, increase the sensitivity of KCs towards their action by upregulating STAT proteins [80, 207].

KCs in psoriasis have potently upregulated inflammatory NF-κB signaling [278]. This pathway is activated by multiple stimuli, including TNF-α, plasmin, and TLRs ligands that are increased in the psoriatic microenvironment [279, 280]. Moreover, several KCs-associated mutations activating the NF-κB pathway were identified in psoriasis patients, including gain-of-function mutations in the CARD14 gene that encodes an activator of NF-κB signaling or loss-of-function in the TNFAIP3 gene that encodes an inhibitor of NF-κB pathway [265].

In inflamed skin, KCs act as nonprofessional antigen-presenting cells (APCs). KCs activated by IFN-γ co-stimulate naïve T cells via CD58/CD2 and to a lesser extent CD54/LFA-1 [192]. Notably, epidermal T cells in psoriatic lesions express CD2 but not CD28, suggesting that interaction of CD58/CD2 may be crucial for T cell activation in the skin [192]. Naïve T cells activated by psoriatic KCs selectively differentiate into T_H_1 and T_H_17 cells [192]. T cells activated by KCs selectively express skin-homing factors, including CCR4 and CCR8 [192]. Moreover, KCs via TGF-β induce CD103 (integrin αE) expression by T cells which is required for immune cell entry into the epidermis [281]. This, together with the fact that activated KCs secrete chemokines that attract IL-17-producing cells, makes KCs key regulators of the maintenance and amplification of immune cells infiltration in psoriatic skin.

### The Role of Fibroblasts

Fibroblasts in the skin create a microenvironment regulating KCs activity, proliferation, and differentiation. In psoriasis, fibroblasts contribute to the pathogenesis of the disease mainly by inducing hyperproliferation of KCs [282]. Insulin-like growth factor-I (IGF-I) is one of the growth factors secreted by fibroblasts promoting KCs proliferation [283]. Moreover, dermal fibroblast of psoriasis patients has increased expression of α5 integrin, fibronectin, keratinocyte growth factor, and fibroblast growth factor receptor 2 that regulate proliferation and differentiation of KCs [284].

Besides the regulation of KCs, fibroblasts also contribute to the regulation of psoriatic inflammation. Fibroblasts in psoriasis have increased expression of IL-6, CXCL2, CXCL12, and CCL19 [164, 285]. Moreover, they produce CXCL8 which is potentiated by TNF-α [286]. Notably, the expression of IL-6 and CXCL8 in activated psoriatic fibroblasts is much higher than in psoriatic KCs [285]. Moreover, fibroblasts triggered by IL-17 promote the production of IL-17 by skin-infiltrating γδ T cells amplifying psoriatic inflammation [119]. Thus, fibroblasts can modulate chemotaxis and activity of neutrophils, T cells, and other types of immune cells in psoriatic skin.

### The Role of Endothelial Cells

One of the histological hallmarks of psoriatic skin is increased dermal vascularity [287]. It is caused by increased concentrations of VEGF, angiopoietin, and TNF-α and is regulated predominantly by psoriatic γδ T cells and T_H_9 cells [48, 184]. Moreover, dermal endothelial cells are activated by psoriasis cytokines, including IL-36γ and IL-17A, which induce their proliferation, secretion of proinflammatory cytokines and chemokines, and upregulate ICAM-1 expression [288]. Transcriptional studies of psoriatic endothelial cells identified enrichment clusters relating to leukocyte adhesion, T cell activation, and IL-8 response in psoriatic lesional skin [164]. Increased angiogenesis and activation of endothelial cells facilitate diapedesis and infiltration of immune cells into the skin amplifying psoriatic inflammation.

## Insights Into the Immune Network in Psoriasis from “Omics” Studies

Advances in research techniques enable more comprehensive studies on multiple types of cells and their interactions contributing to the initiation and progression of psoriasis (Table 3). These global analyses not only confirmed observations described in murine psoriasis models [289] but also revealed a complex network of interaction between immune cells and between immune and non-immune cells and uncovered novel mechanisms regulating psoriatic inflammation.

**Table 3:** High-throughput analysis of the immune landscape of psoriasis

**Reference**	**Samples**	**Number of patients**	**Methodology**	**Main results**
Mehta et al. [163]	Lesional and non-lesional skin	*n = 20*	High-dimensional flow cytometry	- CD11c^+^HLA-DR^+^ myeloid cells, CD64^bright^CD163^−^CD14^bright^CD1c^−^CD1a^−^ inflammatory monocytes are the main source of IL-23
				- CD4^+^CD49a^−^ CD103^−^ T cells and CD8^+^ T_RM_ cells produce IL-17A and are PD-1^+^
				- IL-23p19 and IL-17A inhibitors reduced IL-23^+^ myeloid cells
Guo et al. [290]	Peripheral blood of patients with psoriasis	*n = 38 and 30 HC*	High-dimensional single-cell mass cytometry	- Identification of new cell subsets abundant in psoriasis CD3^–^ CD4^+^ lymphoid tissue inducer cells, Tc17 cells, and CD8^+^ CXCR3^+^ Tregs
				- CD3^−^ CD4^+^ cells have high OX40 levels, decreased FRA2 expression, and correlate with the clinical severity
Farrera et al. [291]	Peripheral blood of patients with psoriasis	*n* = 19 and 9 HC	High-dimensional single-cell mass cytometry	- Decreased frequency of circulating mucosal-associated invariant T (MAIT) cells
				- Increased frequency of circulating memory Treg cells
Fyhrquist et al. [292]	Lesional skin from psoriasis patients	*n* = 134 and 126 HC	cDNA microarrays	- Upregulation of Interferon signaling, LPS-IL-1-mediated inhibition of RXR function, the inflammasome pathway, and T_H_17 signaling
				- Enrichment of leukocyte activation genes
				- Upregulation of inflammatory mediators (S100 proteins, defensins, matrix metalloproteinases, IL-1 family cytokines), T helper-related genes (CCL1, CCL18, IL17A, IL22, PI3/Elafin), barrier genes (KRT16, SERPINB4, KLK9, FLG2, LCE5A, CLDN8), and genes involved in metabolism of tryptophan
Li et al. [28]	Lesional skin from patients with psoriasis	*n* = 92 and 82 HC	Bulk-tissue RNA-seq	- Expression of modules of epidermal differentiation genes
				- Enriched lymphoid and myeloid signature and IL-17-induced genes in KCs
Nattkemper et al. [293]	Lesional and non-lesional skin from patients with psoriasis	*n* = 25 and 30 HC	Bulk-tissue RNA-seq	- Identification of “itchscriptome”—upregulated genes associated with itch intensity (phospholipase A2 IVD, substance P, voltage-gated sodium channel 1.7, and transient receptor potential (TRP) vanilloid 1)
He et al. [294]	Tape strips obtained from lesional and non-lesional skin from patients with psoriasis	*n* = 20 and 20 HC	RNA-seq	- Increased levels of DCs and T cell markers
				- Increased levels of T_H_17-related, T_H_1-related, T_H_22-IL-22-related, and innate immunity-related genes
				- Downregulated levels of markers of terminal differentiation, tight junction, and lipid biosynthesis and metabolism
Tsoi et al. [60]	Lesional and non-lesional skin from patients with psoriasis	*n* = 28 and 38 HC	Bulk-tissue RNA-seq	- Enrichment in the genes associated with immunologic response, IFN, and cytokine signaling pathways
				- Enrichment in the genes associated with MHC class I—antigen processing/presentation, cell-cycle, and arginine/proline metabolism
				- Enrichment in the genes associated with keratinocyte differentiation and cytokine activity in non-lesional skin in psoriatic patients
Tsoi et al. [295]	Lesional and non-lesional skin from patients with psoriasis treated with etanercept	*n* = 42	Bulk-tissue RNA-seq	- The profile of gene expression, including USP18 and KRT2, at baseline of nonlesional psoriatic skin is associated with response to therapy
Swindell et al. [296]	Meta-analysis of RNA-seq performed from lesional and non-lesional skin from patients with psoriasis	*n* = 44	Bulk-tissue RNA-seq	- Induction of psoriasis-specific dysregulated genes by IL-17A
				- Induction of non-specific dysregulated genes by IFN-γ and TNF-α
				- Circulating immune cells share expression signature with other autoimmune diseases
Nakamizo et al. [297]	Cells isolated by fluorescence-activated cell sorting from dissociated skin tissue	*n* = 2 and 2 HC	Index-sorted single-cell flow cytometry and RNA sequencing	- Identification of CD14^+^ type 3 DCs enriched in psoriatic skin and co-producing IL-1B and IL-23A
Gao et al. [298]	Dermis and epidermis of patients with psoriasis vulgaris	*n* = 3 and 3 HC	Single-cell RNA-seq	- Upregulation of MHC complex molecules
				- Upregulation of interferon signaling neutrophil modulation, cytokine, and chemokine production
Kim et al. [299]	Cells isolated by fluorescence-activated cell sorting from dissociated skin tissue	*n* = 1	Single-cell RNA-seq	- Increased frequency of lymphocytes and myeloid cells in relapsing area after anti-IL-17A therapy
				- Enrichment of T_RM_ in relapsing psoriasis
				- Upregulation of T17 signature, lipid metabolism maintaining T_RM_ homeostasis, NF-κB signaling, and CXCL13 in lymphoid cells from relapsing psoriasis
Kim et al. [206]	Cells emigrating from punch biopsy skin	*n* = 13 and 5 HC	Single-cell RNA-seq	- Identification of cutaneous T17 cells
				- Identification of regulatory IL-10-expressing semimature DCs and a subset of semimature DCs expressing IL-23A and IL-36G
				- Impairment of CCL27-CCR10 interaction due to decreased CCL27 expression in basal KCs
Cheng et al. [182]	Cells isolated by fluorescence-activated cell sorting from dissociated skin tissue	*n* = 3 and 3 HC	Single-cell RNA-seq	- Inflammatory response of KCs as well as melanocytes and immune cells
				- High plasticity of cell transcriptional identities of KCs
				- Enrichment of CD1c^+^CD301A^+^ DCs
Zhang et al. [300]	Lesional skin-infiltrating and circulating immune cells	*n* = 10	Single-cell RNA-seq and single-cells TCR-seq	- Clonal expansion of CD8^+^ T_EM_ cells in lesional skin and circulation
				- Strong chemotaxis and cytotoxic signature of T cells in lesional skin
				- Activation of antiviral responses in macrophages
Liu et al. [301]	CD45 + cells isolated from skin	*n* = 8 and 7 HC	Single-cell RNA-seq	- Dysregulation of skin-resident memory T cells
Reynolds et al. [164]	Cells isolated by fluorescence-activated cell sorting from dissociated skin tissue	*n* = 3 and 5 HC	Single-cell RNA-seq of sorted cell populations	- Clonal expansion of disease-associated lymphocytes
				- Reemergence of prenatal vascular endothelial cell and macrophages cellular programs
Liu et al. [218]	Sorted CD8^+^ T cells from lesional skin biopsies	*n* = 11 and 5 HC	Single-cell RNA-seq of sorted CD8^+^ T cells	- Identification of 11 CD8^+^ T cells subset
				- Identification of two non-exhausted Tc17 subsets expressing CXCL13 characterized by upregulated cytokine, cytolytic and metabolic transcriptional activity and associated with disease severity
Roesner et al. [253]	Lesional and peripheral blood T cells	*n* = 10	TCR sequencing of T cells	- T cell repertoires of lesional skin are mirrored by CLA^+^ circulating T cells
				- Identification of frequent T cell clones within CLA^−^ T cells
Harden et al. [254]	Lesional and non-lesional skin from patients or healthy controls	*n* = 8 and 7 HC	High-throughput deep sequencing of TCR	- T cell repertoire in psoriasis is polyclonal

High-dimensional flow cytometry enables the identification of major immune cell types contributing to the pathogenesis of psoriasis as well as their dynamics during therapy. It identified specific myeloid cell populations that are the main producers of IL-23 while T cells are predominantly responsible for IL-17 production [163]. Moreover, it identified a novel population of CD3^–^ CD4^+^ lymphoid tissue inducer cells abundant in psoriatic lesions [290]. Mass cytometry also uncovered the general sequence of the events during psoriasis initiation, progression, and maintenance and the composition of the epidermal immune microenvironment [186].

High-throughput analysis of psoriatic transcriptome confirmed a global character of inflammatory response in KCs, melanocytes, and immune cells [182] and the key role of the IL-17 signaling in inflamed skin [28]. Single-cell RNA sequencing identified subsets of T17 cells [206] and CD8^+^ T cells [218], dysregulation of skin-resident T cells [301], the key population of DCs in psoriatic lesions producing IL-1β and IL-23A [297], and IL-10-expressing regulatory DCs [206]. Moreover, TCR sequencing of T cells of psoriasis patients revealed polyclonal character of immune response and suggested the existence of autoantigens outside the skin [253, 254]. Nonetheless, further mechanistic studies are required to confirm these observations and to dissect the role of newly identified cell populations in psoriasis.

## Conclusions

Exact understanding the mechanisms of psoriasis development and thus the use of effective treatment is important because psoriasis can be associated with distant complications and an even more frequent incidence of lymphohematologic malignancies [302]. The last decades clarified the view of psoriasis as a T_H_17 disease with the important components of T_H_1 and T_H_22 response and revealed crucial dysregulated components leading to the development of the disease. Emerging evidence demonstrated a key role of IL-17, IL-23, and TNF-α and resulted in the development and approval of biological therapies that revolutionized psoriasis management. It is well established that T cells orchestrate psoriatic inflammation with the help of multiple cells, including DCs, neutrophils, and KCs. Moreover, the role of several types of cells, including regulatory B and T cells, or autoantibodies in psoriasis remains largely unknown. Moreover, the role of the recently identified immunoregulatory population of cells named CD71^+^ erythroid cells (CECs) in psoriasis remains unknown [303, 304]. Recently, there has been a growing awareness of the role of the microbiota in the regulation of immunity. Indeed, growing evidence suggests the role of impaired skin and gut microbiome composition in the pathogenesis of psoriasis [292, 305]. Our understanding of the dysregulation of immune response in psoriasis has improved significantly over the past decade which offers the potential for the further development of even more effective and durable therapeutic strategies.

## Data Availability

No datasets were generated or analysed during the current study.
